# Thoracic Cavernous Lymphangioma Provoking Massive Chyloptysis: A Case Report

**DOI:** 10.1177/2324709613503315

**Published:** 2013-09-11

**Authors:** Robert Ferguson, Jeffrey Hodges, Cayce Harness-Brumley, Carlos Girod, Sonja Bartolome, J. Michael DiMaio

**Affiliations:** 1 University of Texas Southwestern, Dallas, TX, USA

**Keywords:** mediastinal lymphangioma, cardiopulmonary bypass, chyloptysis

## Abstract

Chyloptysis is a relatively rare embodiment of disease that encompasses a lengthy differential and provides many diagnostic and therapeutic challenges. Presented here is the case of a young woman with massive chyloptysis due to a thoracic cavernous lymphangioma arising in the peripartum period. The severity of her condition mandated the use of cardiopulmonary bypass to resect her lymphangioma. We believe that the extent of her symptoms, etiology of disease, and surgical management represent a unique scenario in the literature.

## Introduction

Lymphangiomas account for up to 4.5% of mediastinal tumors.^1,2^ Its embryologic origin is the aberrant proliferation or outgrowth of tissues derived from the primitive jugular sac. The overwhelming majority arise during fetal development and childhood but there have been rare reported cases of such structures being unveiled later in life.^2^ The most widely accepted classification includes (*a*) cystic lymphangioma (ie, cystic hygroma), (*b*) cavernous lymphangioma, and (*c*) lymphangioma simplex. Largely asymptomatic, cavernous lymphangioma can present a diagnostic challenge often requiring lymphoscintigraphy and magnetic resonance imaging (MRI) to identify. Once recognized and treated with surgical resection, as in our case, the long-term prognosis is excellent.

## Case Report

**Figure 1.**
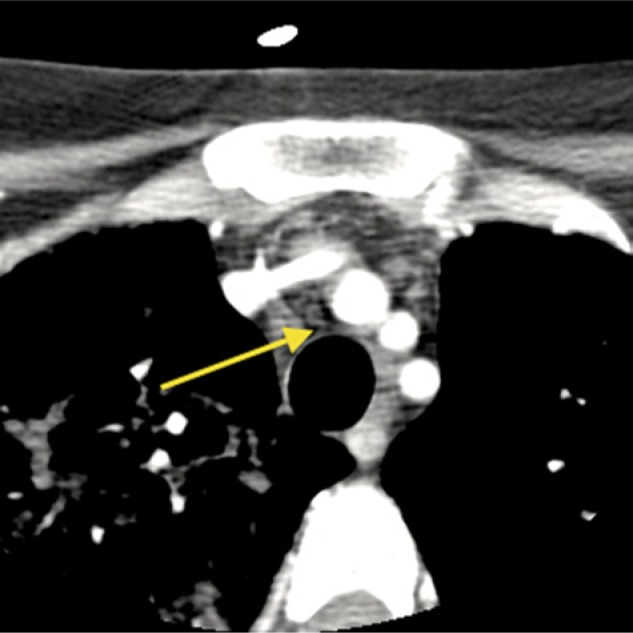
Computed tomography scan of the neck: The arrow indicates a soft tissue between the trachea and innominate artery.

**Figure 2.**
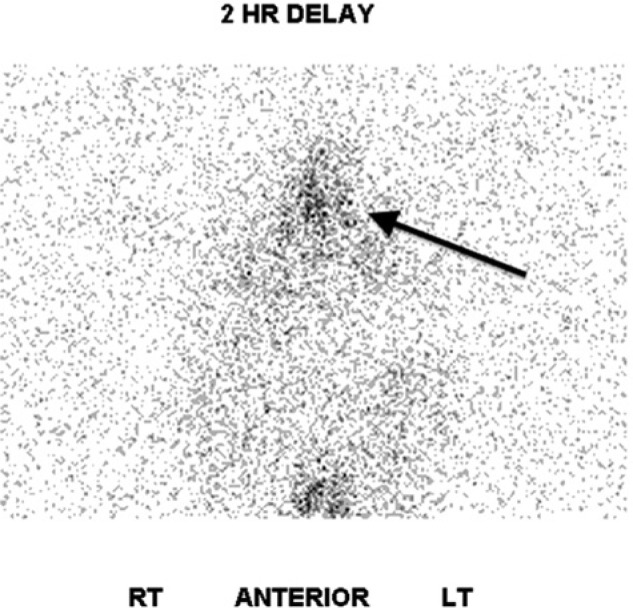
Lymphoscintigraphy: The arrow indicates clustering of tracer uptake within the mediastinum.

A 26-year-old Hispanic lady without antecedent history was admitted to the intensive care unit with acute respiratory failure and massive chyloptysis requiring mechanical ventilation. Two years prior she began to experience a persistent, frothy white productive cough, dyspnea, and fatigue following an uncomplicated caesarean section. Various pulmonary conditions, including recurrent community-acquired pneumonia, pulmonary alveolar proteinosis, granulomatous inflammation, and bronchial anthracofibrosis had been entertained and a VATS (video-assisted thoracoscopic surgery) lung biopsy revealed nodular perivascular anthracosis with chronic inflammation and local fibrosis. She had developed bilateral chylous effusions (right greater than left) and parenchymal consolidations. Infectious etiologies were meticulously ruled out. Chemical analysis of her sputum revealed a triglyceride level of 251 mg/dL, a cholesterol of 8 mg/dL, bilirubin <1 µg/mL, hemoglobin of 6 µg/mL, and the presence of a large quantity of chylomicrons. A lymphatic disorder was suspected and she underwent an exhaustive workup, including a high-resolution computed tomography (CT) scan of the head/neck/chest as well as an I-125 albumin lymphoscintigraphy (Figures 1 and 2). The workup failed to identify the etiology of her chylous output. MRI was considered but was forgone given her critically ill condition and ventilator status. As she continued to deteriorate more extensive upper and lower extremity lymphoscintigraphies were performed which suggested a disruption of the lymphatic circulation at the level of the thoracic cavity but could not identify a definitive leak.

She was taken to the operating room and underwent thoracic duct ligation via a minimally invasive approach through the right hemithorax with the intent of treating an underlying thoracic lymphangiectasis. After a transient improvement in her condition and in her chylous output, she deteriorated rapidly and experienced multisystem organ failure and the decision was made to return to the operating room for a more extensive exploration. Given her circulatory collapse and hypoxemia, she could not tolerate single-lung ventilation. In addition, the planned diagnostic and therapeutic bronchoscopy would preclude the use of a double lumen tube and require prolonged periods of imperfect to inadequate ventilation. With this in mind and to provide optimal exposure to the proposed operative field, she underwent median sternotomy and was placed on cardiopulmonary bypass.

**Figure 3.**
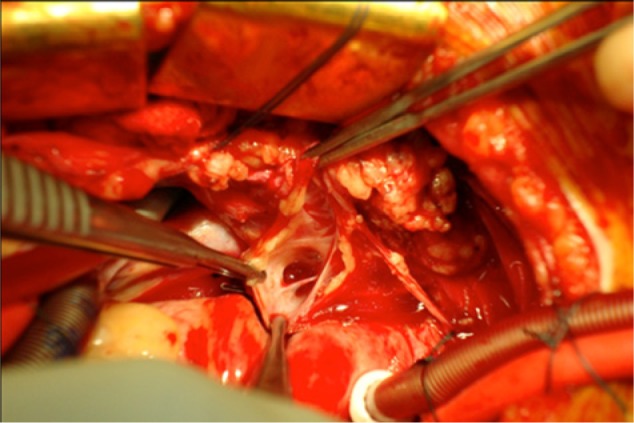
Opening the communication of lymph canal on cardiopulmonary bypass.

**Figure 4.**
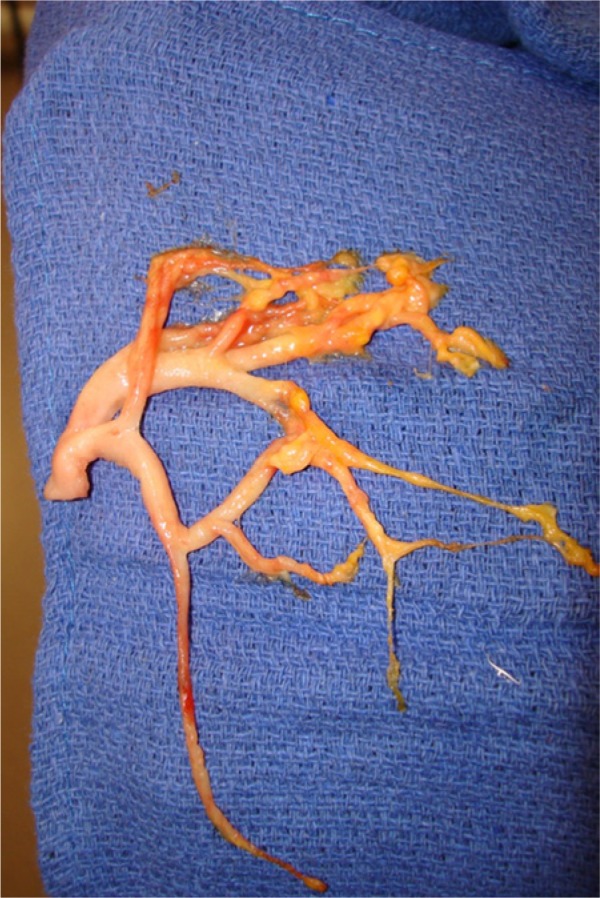
Chylous cast of the bronchial tree removed intraoperatively.

Dissection of the mediastinum revealed a singular lymphatic structure arising near the take-off of the right carotid artery. It coursed posterior to the innominate vessels and branched above the right pulmonary artery with tributaries extending toward the right and left main stem bronchi. There was communication with the right middle and lower lobes (Figure 3) and no evidence of communication with the left bronchi confirmed by injection of dye. This lymphatic structure was ligated at its proximal origin and resected. Additionally, given the diffuse parenchymal damage to the right lung, the immense size of the fistulae encountered, and concern for postoperative recannalization or breakdown, a right-sided bilobectomy was performed sparing the upper lobe. A large flap of pericardial fat and parietal pleural was used to buttress the bronchial stump given the poor quality of the diseased and inflammed tissue. Concomitantly, a bronchoscopy was performed extracting extensive chylous casts from both pulmonary trees (Figure 4). She made an impressive postoperative recovery and was discharged home for rehabilitation with abrogation of her respiratory symptoms. At 18-month follow-up, she remains symptom free and disease free as evidenced by a 12-month follow-up bronchoscopy. The use of CT scan for follow-up surveillance has been entertained; however, given the fact that it did not provide a clear diagnosis initially, it remains unclear if it will provide any benefit above tracking her clinical progress.

**Figure 5.**
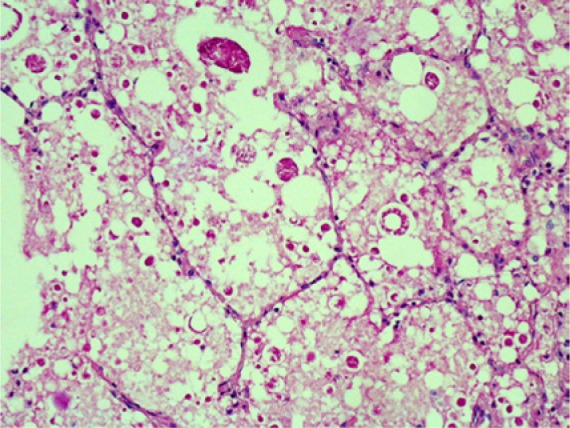
Periodic acid–Schiff stain of our patient’s lung. Notice the large interspersed vacuoles of the chylous deposits.

**Figure 6.**
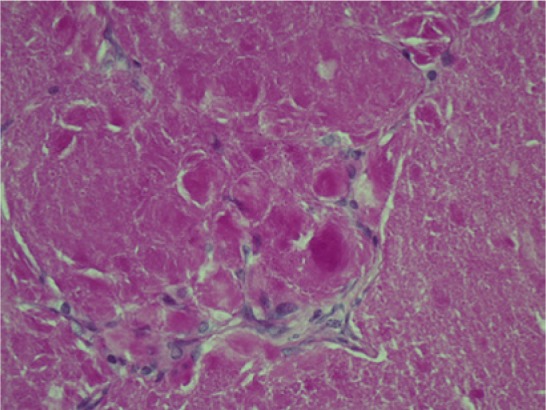
Periodic acid–Schiff stain of a patient with pulmonary alveolar proteinosis. Notice the dense protein deposits and absence of the vacuoles as noted in our patient.

**Figure 7.**
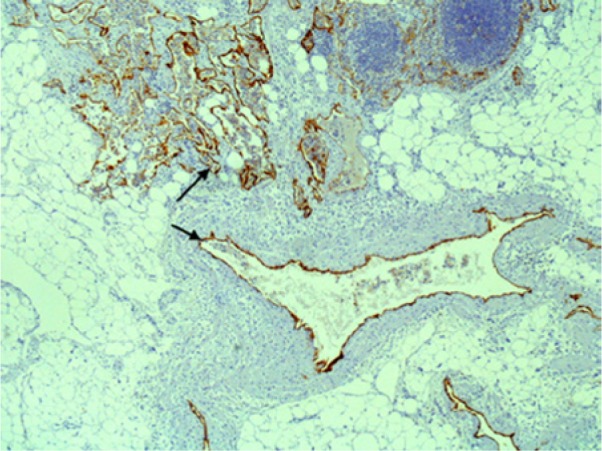
Low-power D2-40-positive lymphatic channels. Arrow indicates the staining specific to lymphatic endothelium.

**Figure 8.**
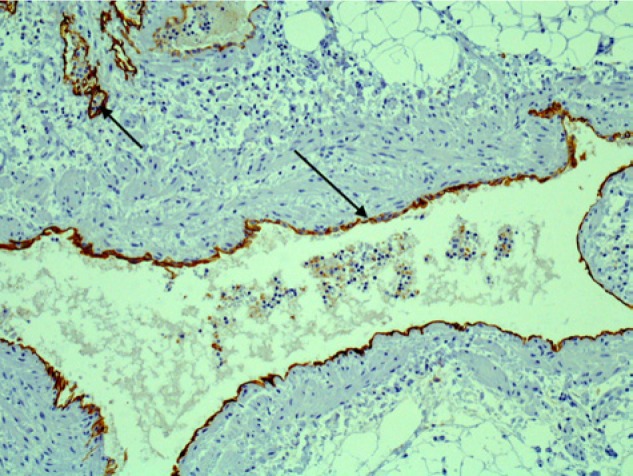
High-power D2-40-positive lymphatic channels.

Interpretation of the specimens by anatomic pathology described the lung parenchyma as having been nearly replaced by chyle-filled alveolar spaces consisting of lipoid material. Periodic acid–Schiff staining was negative thus ruling out pulmonary alveolar proteinosis. Further histopathologic investigation ascertained the presence of an abnormal proliferation of enlarged vascular channels lined with endothelium. Positive staining by D2-40 of these endothelial cells affirmed that these channels were indeed lymphatic proliferations (Figures 5-8).

## Discussion

Cavernous lymphangioma usually originates in the head and neck or axilla and is characterized by its isolated location and benign proliferation as opposed to lymphagiomatosis, which is more aggressive, arises in multiple anatomic locations and is a more common cause of chyloptysis.

Chyloptysis is a rare manifestation of lymphangioma. It is more typical of other lymphatic disorders such as the aforementioned lymphangiomatosis, lymphangioleiomyomatosis, thoracic lymphangiectasis, yellow nail syndrome, and iatrogenic injury.^3^ In our review of the literature there were infrequent citations of this association and no series of patients with thoracic cavernous lymphangioma manifesting as chyloptysis. The largest and most important series of 25 lymphangiomas from the Mayo Clinic did not identify chyloptysis as a presenting symptom.^1^ Likewise, the use of cardiopulmonary bypass in this exigent setting represents a novel report in the literature. There have been case reports describing the use of cardiopulmonary bypass for the resection of primary cardiac cystic lymphangiomas but not for the resection of a primary thoracic cavernous lymphangioma.^4^

The frustrating aspect of our case was the difficulty in diagnosis of the source of chyloptysis and localization of this lady’s lymphangioma. Lymphoscintigraphy has been the historical, noninvasive, imaging modality of choice. It is limited, however, by its low specificity and inability to pinpoint individual anatomic structures. More recently high resolution CT scan and MRI have best been used to diagnosis mediastinal lymphangiomas although most authors admit that radiographic diagnosis alone remains challenging and elusive.^5^ MRI may have identified a characteristic high-intensity lesion on T2-weighted images.

As to the etiology of the patient’s disease, previous case reports have described lymphangiomas that arise during pregnancy. There have been 2 cases of axillary lymphangioma occurring in the third trimester and a single case of abdominal lymphangioma discovered during the 14th week.^6^ We believe this is the first documented case of a peripartum thoracic cavernous lymphangioma. Quack Loetscher et al^6^ postulated that this phenomenon is likely due to the presence of increased vessel-inducing factors, for example, vascular endothelial growth factor, during the course of pregnancy. We believe that our case fits the pattern previously described given the onset of chyloptysis following her caesarian section.

Although a benign disease, most thoracic lymphangiomas that come to light will become symptomatic over time and the treatment of choice is surgical resection. Complete resection portends an excellent prognosis with equivalent life expectancy in age matched populations. There have been reports of up to a 30% recurrence rate but given the paucity of large series, resection should remain the mainstay of treatment. With the presentation of chyloptysis, it is imperative to recognize a lymphatic disorder or injury and proceed with timely surgical intervention as necessary and not be forced to intervene under duress.
